# Developmental Plasticity in Butterfly Eyespot Mutants: Variation in Thermal Reaction Norms across Genotypes and Pigmentation Traits

**DOI:** 10.3390/insects13111000

**Published:** 2022-10-31

**Authors:** Ana Rita Amaro Mateus, Patrícia Beldade

**Affiliations:** 1 Instituto Gulbenkian de Ciência (IGC), 2780-156 Oeiras, Portugal; 2 CNRS—UMR 5174, Evolution et Diversité Biologique (EDB), Université Paul Sabatier (UPS), 31077 Toulouse, France; 3 Center for Ecology, Evolution and Environmental Changes (cE3c) & Global Change and Sustainability Institute (CHANGE), Faculty of Sciences, University of Lisbon (FCUL), 1749-016 Lisbon, Portugal

**Keywords:** developmental plasticity, seasonal polyphenism, pigmentation mutants, *Bicyclus anynana*, butterfly eyespots, gene-by-environment interactions, thermal reaction norms, trait integration, trait-specific effects

## Abstract

**Simple Summary:**

The temperature experienced during organismal development can affect adult phenotypes. This phenomenon is called thermal developmental plasticity and is common for insect pigmentation. Plasticity can help organisms cope with environmental heterogeneity, such as that across yearly seasons, and might also impact how populations deal with environmental perturbation, such as climate change. Because plasticity can be adaptive and heritable, it is liable to evolve. The evolution of plasticity will depend on the availability of genetic variation contributing to variation in plasticity, and on the integration across plastic traits, which might limit opportunities for their independent evolutionary change. Here we address these two topics by focusing on thermal plasticity in butterfly wing pattern elements called eyespots, which are composed of concentric rings of different colors. We found differences in plasticity between genotypes and concluded that mutants of strong effect on pigmentation can also affect thermal plasticity therein. We also found differences in plasticity between distinct pigmentation features, suggesting plasticity can evolve somewhat independently for those features.

**Abstract:**

Developmental plasticity refers to the property by which a genotype corresponds to distinct phenotypes depending on the environmental conditions experienced during development. This dependence of phenotype expression on environment is graphically represented by reaction norms, which can differ between traits and between genotypes. Even though genetic variation for reaction norms provides the basis for the evolution of plasticity, we know little about the genes that contribute to that variation. This includes understanding to what extent those are the same genes that contribute to inter-individual variation in a fixed environment. Here, we quantified thermal plasticity in butterfly lines that differ in pigmentation phenotype to test the hypothesis that alleles affecting pigmentation also affect plasticity therein. We characterized thermal reaction norms for eyespot color rings of distinct *Bicyclus anynana* genetic backgrounds, corresponding to allelic variants affecting eyespot size and color composition. Our results reveal genetic variation for the slope and curvature of reaction norms, with differences between eyespots and between eyespot color rings, as well as between sexes. Our report of prevalent temperature-dependent and compartment-specific allelic effects underscores the complexity of genotype-by-environment interactions and their consequence for the evolution of developmental plasticity.

## 1. Introduction

Developmental plasticity refers to the property by which the same genotype results in distinct phenotypes depending on the environmental conditions experienced during development (reviewed in [1,2,3]). There are many examples of this environmental sensitivity of development, including different types of environmental triggers and various types of plastic traits. For example, nutrition largely determines whether bee and ant larvae develop into workers or queens, which differ in morphological, life-history, and behavioral traits; the density of conspecifics determines whether *Schistocerca* nymphs produce gregarious or solitary locusts, which also differ in body pigmentation (reviewed in [4]); and temperature and/or photoperiod affect wing pigmentation patterns in various butterfly species, which can also differ in physiology and life histories (reviewed in [5,6,7]). In some cases, when the triggering environmental cue is predictive of future environmental conditions, developmental plasticity can result in a better match between the organism’s phenotype and the conditions it will live in. As such, this plasticity can help organisms cope with environmental heterogeneity and be adaptive (reviewed in [2]). This is particularly compelling in the case of seasonal polyphenisms, corresponding to the production of distinct discrete phenotypes adjusted to the conditions of different seasons [5,6,7,8].

Studies on a variety of insect species have provided valuable insight into the ecological significance and the mechanistic underpinnings of plasticity (e.g., [7,9,10,11]), discussed how plasticity may impact evolutionary diversification [12] and resilience to environmental perturbation [13], and discussed the origin and evolution of plasticity (e.g., [14,15,16]). Towards the latter topic, it is important to consider: (1) the genetic variation for plasticity, which provides raw material for selection to act on (e.g., [17,18]), and (2) the integration between co-affected plastic traits, which may condition their independent evolution (e.g., [19]). Central to these studies is the concept of reaction norms (RNs) [20], a graphical representation of plasticity in which phenotype is displayed as a function of environmental conditions. Reaction norms can differ in intercept, slope, and curvature (cf. [21]) depending on the environmental cue and trait considered, as well as on genotype (reviewed in [18]). Heritable variation for reaction norms can fuel the evolution of plasticity, of which there are various examples in natural and experimental populations [14,21,22,23,24,25,26].

Despite great progress in the dissection of the genetic basis of variation and evolutionary change in many adaptive traits (compiled in [27]), not much is known about the genetic basis of variation in reaction norms. Are there “genes for plasticity” [28]? Which genes contribute to inter-genotype differences in plasticity (e.g., [17])? To what extent are those the same genes that contribute to inter-individual variation in trait values within a fixed environment (see [18])? These questions relate to asking about environment-specific allelic effects, or about genotype-by-environment (GxE) effects. Here, we characterized thermal reaction norms for spontaneous pigmentation mutants to assess the extent of GxE interactions and to test the hypothesis that alleles affecting pigmentation also affect plasticity therein. By investigating distinct pigmentation components of multiple serially repeated color pattern elements, we further tested the extent of trait-specific, or spatial compartmentalization of, plasticity. The integration between plastic traits is fundamental for ensuring coherent whole-body phenotypes and can condition their independent evolution.

We focused on an evolutionary ecology model of developmental plasticity, seasonal polyphenism in *Bicyclus anynana* wing pattern ornaments called eyespots (reviewed in [5,29,30,31,32]). *B. anynana* butterflies have eyespots along the margins of their wings, each composed of a central white focus, a middle black ring, and an external golden ring. In early pupae, presumptive eyespot foci produce a substance that diffuses away to create a concentration gradient, which informs neighboring epidermal cells about their position relative to the focus. These cells thereby become fated to produce the different color pigments that make up eyespot rings (recently reviewed in [31]). The focal signal strength and the epidermal response sensitivity to that signal determine eyespot size [5], which can vary also depending on developmental temperature (e.g., [22]).

Studies of thermal plasticity in *B. anynana* eyespots combine analyses of the mechanisms regulating eyespot plasticity with knowledge about the ecological significance and evolution of that plasticity (different aspects reviewed in [5,13,29,30,31,33]). In natural populations of *B. anynana*, plasticity in ventral wing patterns is associated with distinct seasonal strategies to avoid predation [34]. While the larger and brighter eyespots of wet-season butterflies are thought to attract predators’ attention to the wing margin and away from the vulnerable body, the mostly brown dry-season butterflies, with inconspicuous eyespots, are cryptic against the background of dry leaves characteristic of that season [33,35,36,37]. In the laboratory, ambient temperature during development determines the production of alternative phenotypes resembling the natural wet and dry seasonal forms [29]. While both temperature (T)- and genotype (G)-based variations in *B. anynana* eyespot size have been relatively well characterized (e.g., [29,38,39,40,41,42,43,44,45]), much less is known about genotype-by-temperature (GxT) interactions.

Here, we focus on spontaneous mutant alleles of large effect on eyespot morphology to ask about GxT effects and test the hypothesis that alleles that contribute to variation in pigmentation (within temperatures) also contribute to variation in levels of pigmentation plasticity. We do this by characterizing thermal reaction norms for the size of eyespot color rings for *B. anynana* mutants with altered eyespot size or color composition. Looking at different aspects of eyespot patterns, including different individual eyespots on the same and distinct wings, as well as different color rings of individual eyespots allows us to assess spatial compartmentalization or cross-trait interaction of GxT effects. We show evidence for pervasive GxT effects, with differences in reaction norms between pigmentation mutants, indicating that pigmentation genes can also affect thermal plasticity in pigmentation. We also show that reaction norms differ between color rings and between individual eyespots, in a manner that can vary between the sexes.

## 2. Materials and Methods

### 2.1. Animals

**Figure 1:**
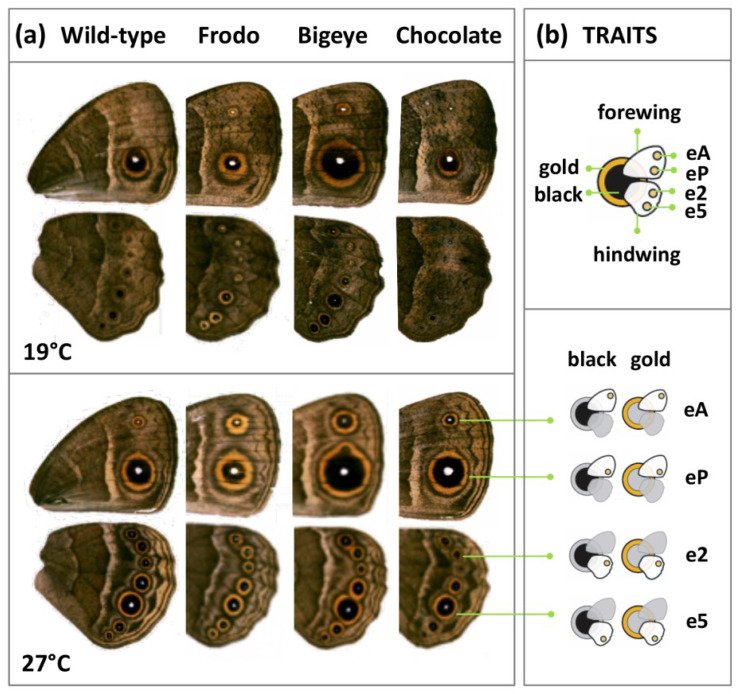
Wing traits measured in adult butterflies from four genotypes. (**a**) The photos represent the typical phenotype of ventral wing surfaces of *Bicyclus anynana* females from four genetic stocks (WT, Fr, BE, and Choc) reared at 19 °C (top panel) or 27 °C (bottom panel). For each individual, we obtained measurements of the areas of the black and golden rings of two eyespots on the forewing (eA and eP, for the anterior and posterior eyespots, respectively) and two on the hindwing (e2 and e5, for the second and fifth eyespots, respectively), as well as forewing and hindwing areas. (**b**) The diagram on top displays the symbols used to represent the different traits we measured. For each of the two eyespots measured on each wing, the more anterior is represented by a circle on the top of the wing, and the more posterior by a circle on the bottom of the wing. The color of the circles at the center of each icon corresponds to either the black or golden rings.

We used captive populations of *Bicyclus anynana* butterflies with different pigmentation phenotypes (Figure 1): an outbred stock representing the “wild-type” phenotype (WT, [29]); a larval color mutant with wild-type adult pigmentation, called Chocolate (Choc, [46]); and two eyespot mutants, Bigeye (BE), affecting eyespot size, and Frodo (Fr), affecting eyespot color composition [47]. While the Choc stock is pure-breeding for the mutant allele, BE and Fr stocks always segregate for mutant and wild-type-looking individuals, as the respective causative alleles are recessive embryonic lethal [47]. Individuals heterozygous for the BE mutant allele have overall enlarged eyespots, and individuals heterozygous for the Fr mutant allele have a higher golden-to-black ratio. We use the term “genotype” to refer to each of the four stocks differing in pigmentation phenotype, even though there is genetic variation within stocks, i.e., they are not isogenic.

About 120 first-instar larvae from each stock were transferred into each of three climate-controlled rooms (70% relative humidity, 12:12 h light:dark cycle) differing in ambient temperature (±0.5 °C). We chose temperatures typically used to induce experimentally the natural dry (19 °C) and wet (27 °C) seasonal morphs, and an intermediate temperature (23 °C). Larvae were kept in large cages and fed ad libitum with young maize plants sprayed with anti-fungal solution. Adults were frozen 24 h after eclosion, and their wings were cut and stored in the freezer until analysis. Due to a microsporidial infection in all laboratory stocks, we obtained fewer than 120 adults per stock per temperature. Final sample sizes are provided in supplementary File S2.

### 2.2. Pigmentation Traits

The ventral surfaces of the undamaged right forewing and hindwing of adult females and males were photographed (Leica DC200 digital camera) under a binocular microscope (Leica MZ12) at 10× magnification. This was performed under controlled light conditions, using the support of a ruler for conversion from pixels to millimeters and a color reference card (QPcard 201) for color calibration and background correction. The resulting images were analyzed with a custom image processing system (cf. [43]) using the ImageJ-based open-source Fiji software package [48]. Briefly, areas of eyespot color rings were calculated by a threshold method in which the image was converted to black and white, the values of intensity under or above user-established thresholds were selected and user-validated, and corresponding areas were determined. Each of these areas corresponds to one color ring and excludes other color rings inside it. In total, we measured eight areas characterizing eyespot color rings (Figure 1), as well as the total areas of both fore- and hindwing. The eight eyespot traits correspond to the middle black ring and external golden ring of the two eyespots on the ventral surface of the forewing (the anterior eyespot, eA, and the posterior eyespot, eP), as well as of two of the seven eyespots on the ventral surface of the hindwing (the second and the fifth eyespots, e2 and e5, respectively, corresponding to the equivalent positions of eA and eP, cf. wing venation). This selection includes representatives of the anterior and posterior compartments of both wings (cf. [49]), as well as the eyespot typically targeted in studies of thermal plasticity in *B. anynana*, e5 (e.g., [16,43,50,51,52]).

### 2.3. Statistical Analyses

All data analyses were performed in *R* [53], separately for females and males, already known to differ in wing size and pigmentation. In all statistical models, we used “genotype” to refer to the different genetic backgrounds. We tested for the impact of temperature (T), genotype (G), and their interaction (GxT) on different plastic traits. We considered *temperature* (three levels: 19 °C, 23 °C, and 27 °C) and *genotype* (four levels: WT, Fr, BE, and Choc) as fixed factors in general linear models assuming a Gaussian distribution of errors. Prior to that, parametric assumptions were considered by checking for normality (Shapiro–Wilk test, alpha = 0.05) and homoscedasticity (Fligner–Killeen test, alpha = 0.05) of residuals, and transforming data where appropriate. When a significant difference (alpha = 0.05) was found for the effects of G and/or T, we performed post hoc pairwise comparisons using Tukey’s honest significant difference (HSD) tests (alpha = 0.01).

First, for each of our eight target eyespot traits (Figure 1), we tested for differences between temperatures and genotypes and for the interactions between these two factors. We used the model ring_area~wing_area + temperature*genotype, with *temperature* (three levels: 19 °C, 23 °C, 27 °C) and *genotype* (four levels: WT, Choc, BE, Fr) as fixed factors and *wing_area* as a covariate. Second, we used principal component analysis (PCA) [54] to reduce and explore the patterns of variation for the eight eyespot rings. In order to be able to handle missing values, we used the R packages FactoMineR [55] and missMDA [56]. PCA was run using the values of eyespot ring area/wing area. We stored and represented graphically the scores for the first four principal components, hereafter referred to as dimensions (Dims; terminology in agreement with the R package we used). We then characterized the reaction norms for each of these Dims and tested the model Dim~temperature*genotype, with *temperature* and *genotype* as fixed factors.

Finally, we compared wild-type-looking and mutant-looking individuals that segregate within each of the BE and Fr stocks. Note that we did not include the wild-type-looking individuals from the BE and Fr stocks in the previous analyses. We tested the model ring_area~wing_area + temperature*phenotype using a general linear model with a Gaussian distribution of error. This was performed for the BE and Fr stocks separately, with *temperature* (three levels: 19 °C, 23 °C, 27 °C) and *phenotype* (two levels: mutant, wild type) as fixed factors and *wing_area* as a covariate.

## 3. Results

**Figure 2:**
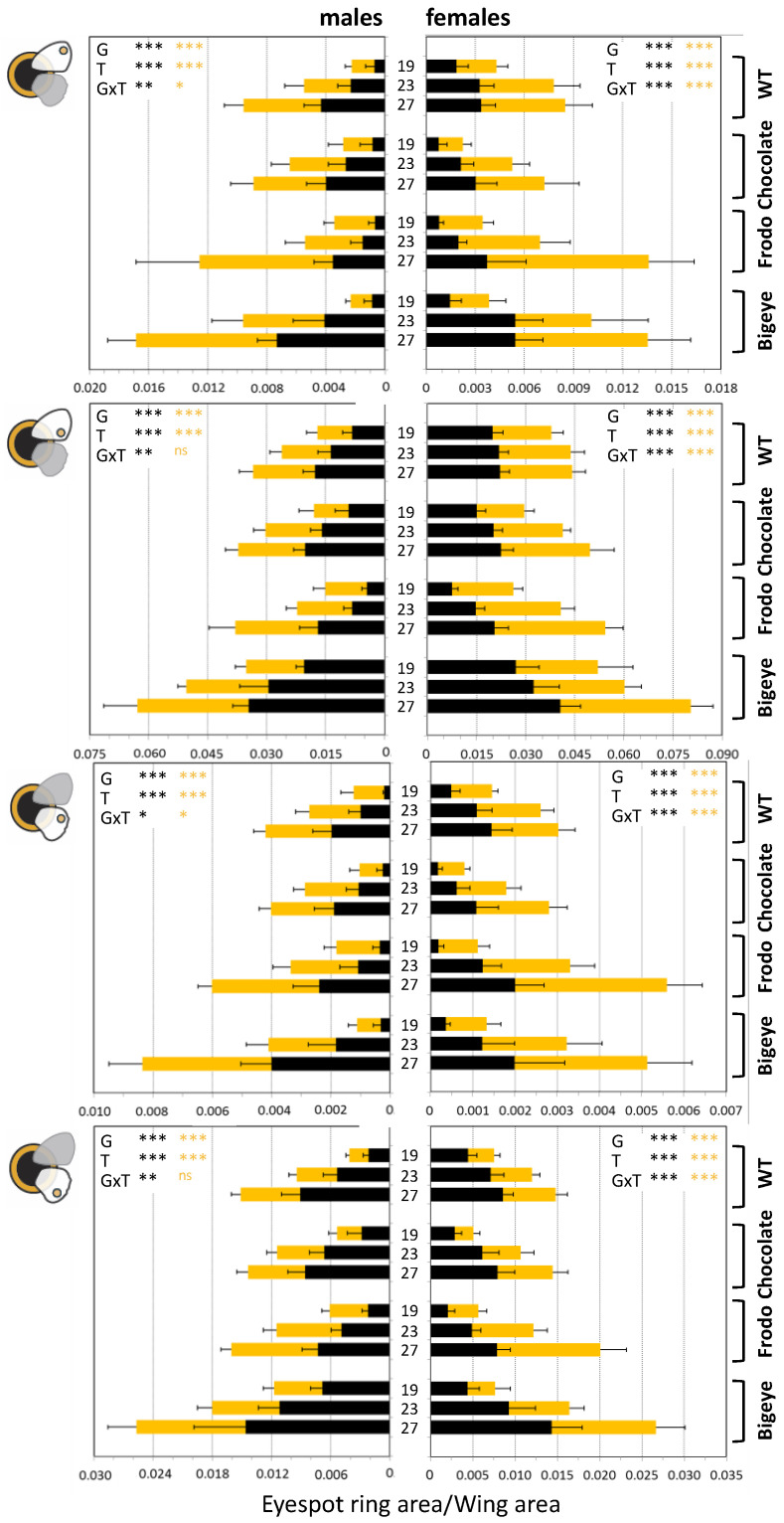
Variation in eyespot rings in relation to developmental temperature and genotype. Areas of the black and golden rings (different colors) of each of the four target eyespots (symbols to the left; see Figure 1) in male (left) and female (right) butterflies from each of the four target genotypes (names to the right) developed at different temperatures (19, 23, or 27 °C). Values correspond to the mean and standard deviation of eyespot area relative to the area of the corresponding wing. We tested for the effect of temperature (T) and genotype (G) on ring area, using wing area as a covariate (see Section 2). Statistical significance of effects of G, T, and GxT for each ring area (different colors) is represented with ^ns^ for non-significant (*p* > 0.05), * for *p* < 0.05, ** for *p* < 0.01, and *** for *p* < 0.001. Raw data in supplementary File S1 and details of the statistical analysis in supplementary File S2.

**Figure 3:**
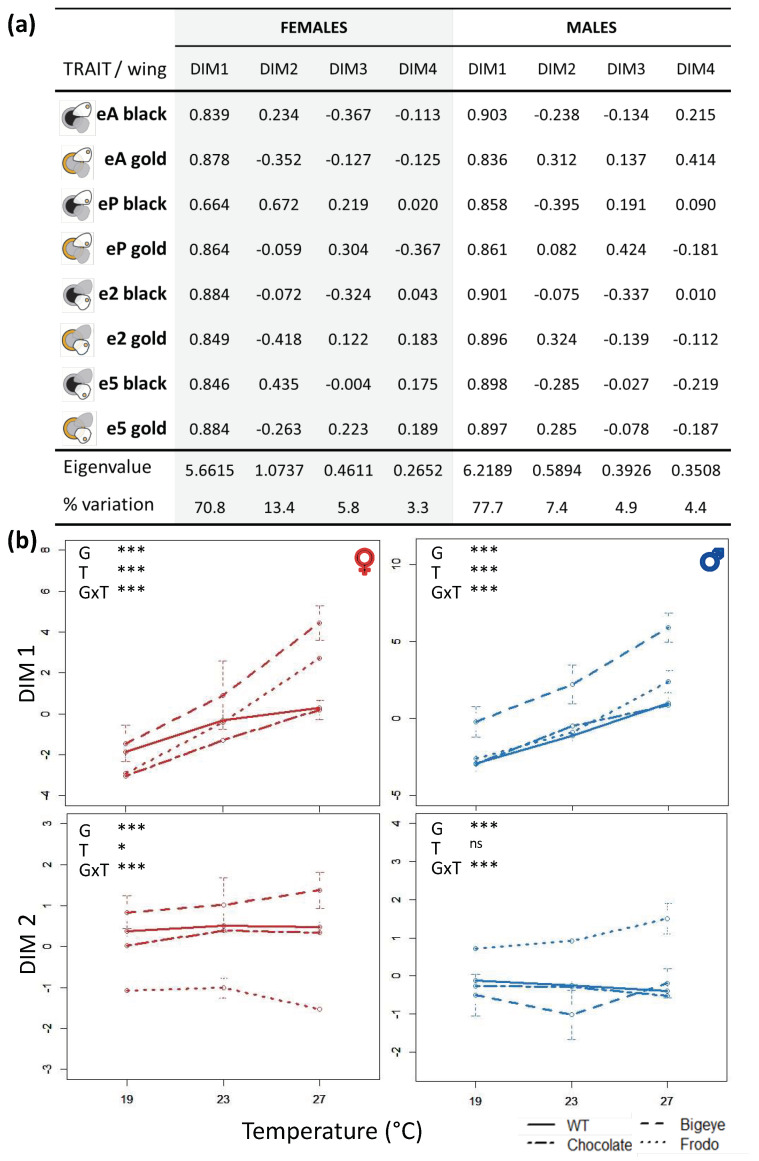
Principal component analysis (PCA) of eyespot ring area for different temperatures and genotypes. (**a**) PCA loadings for each of the eight pigmentation traits (two color rings for each of four target eyespots; symbols on the left), reflecting their contribution to defining the first four principal components identified (termed Dim 1–4; with eigenvalues and % variation explained). The analysis was performed separately for the female and male datasets. (**b**) Mean values along Dim 1 and Dim 2 (with bars representing standard deviation) as a function of developmental temperature (19, 23, or 27°C) for each of the four genotypes (WT, Fr, BE, and Choc in different line styles; legend in bottom right corner) in females (red) and males (blue). We tested for the effect of temperature (T) and genotype (G) on each Dim (see Section 2). Statistical significance of effects of G, T, and GxT for each Dim is represented with ^ns^ for non-significant (*p* > 0.05), * for *p* < 0.05, *** *p* < 0.001. Further details in supplementary File S3.

**Figure 4:**
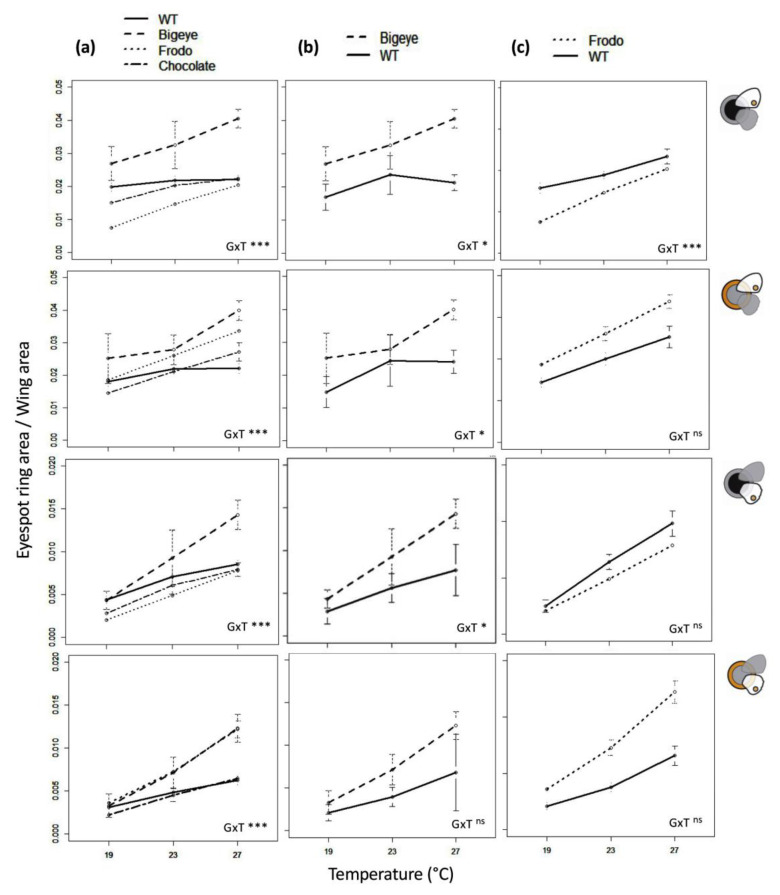
Thermal reaction norms for eyespot ring area for butterflies differing at specific pigmentation loci. Means and standard deviations for eyespot ring areas (symbols to the right) relative to the corresponding wing areas as a function of developmental temperatures in females from different genetic lines (**a**), and for allelic variants for the BE (**b**) and Fr (**c**) lines. For BE and Fr, we compare sibling “mutant” (heterozygous at the respective locus) and “wild type” (homozygous for the wild-type allele, represented by WT and solid line in the figures). We tested for the effect of temperature (T) and genotype (G) on eyespot ring area using wing area as a covariate (see Section 2). Statistical significance for GxT interactions displayed as: ^ns^ (non-significant) *p* > 0.05, * *p* < 0.05, *** *p* < 0.001. Reaction norms for males in File S4, details of statistical analysis in Files S2 (for (**a**)) and S4 (for (**b**,**c**)), raw data in File S1.

To quantify eyespot thermal plasticity in *B. anynana* pigmentation variants, we collected phenotypic data from individuals of four different genetic backgrounds reared at three developmental temperatures (Figure 1; data in File S1). We compared eyespot ring area between genotypes and rearing temperatures (Figure 2 and File S2), and we compared thermal reaction norms for the main principal components defined by our eyespot measurements (Figure 3 and File S3). For the two lines carrying alleles of large effect on eyespot morphology, we compared thermal reaction norms between sibling wild-type-looking (homozygous for wild-type allele) and mutant-looking individuals (heterozygous for mutant allele) (Figure 4 and File S4). Our analysis probed the effects of temperature (T), genetic line (G), and their interaction (GxT) on phenotype. Significant T effects reveal that traits are thermally plastic, significant G effects reveal differences between genetic backgrounds, and significant GxT effects reflect differences between genetic variants (either lines or allelic variants within stocks) in their thermal reaction norms.

### 3.1. Genotype, Temperature, and Genotype-by-Temperature Effects on Eyespot Color Rings

We investigated temperature-induced variation for the area of the golden and black rings of the anterior and posterior eyespots on the forewing (eA and eP) and for the two eyespots on equivalent positions on the hindwing (e2 and e5) in four genetic lines (Figure 1). We observed extensive variation in total eyespot size and size of their color rings, with differences across genotypes (with BE having larger eyespots and Fr having relatively larger golden relative to black rings) and temperatures (with larger color rings with increasing temperatures), but also between individual eyespots (with e2 typically being the smallest and eP the largest) and between sexes (typically larger in females relative to males). There were significant G and T effects for all traits, and significant GxT effects for all but the golden rings of the posterior eyespots (eP and e5) in males (Figure 2 and supplementary File S2).

In Figure 2, connecting the heights of the three adjacent histogram bars, relative to the three experimental temperatures, effectively represents the thermal reaction norm for each trait. We observed differences between genotypes in reaction norm intercept (reflecting differences in trait values within temperatures, e.g., black ring in eP and e5 in males), slope (corresponding to differences in trait values across consecutive temperature values, e.g., black ring in e5 in females), and shape (corresponding to where phenotypes at 23 °C stand relative to the two extreme temperatures, e.g., female eA black 19 > 23~27 while eA gold 19 > 23 > 27). Globally, and for both sexes, BE individuals showed the most pronounced differences between temperatures (i.e., steepest reaction norms), with eyespot ring areas differing the most from wild-type-looking eyespots at 27 °C. BE reaction norms also stood out for the intercept, notably for the black ring, larger than those of wild-type butterflies.

### 3.2. Principal Component Analysis

The PCA describing the patterns of variation for the eight eyespot traits (4 eyespots × 2 color rings) in butterflies from our four target genetic stocks reared at three temperatures reduced variation to four main Dims, together accounting for about 94% of the variation in our female and male datasets (Figure 3). The loadings for the different eyespot traits revealed how each of them contributed to defining each of the Dims (Figure 3a): high absolute values versus values close to zero reflecting high versus low contribution, and positive versus negative values reflecting contrasting contributions. The thermal reaction norms for the main Dims (Figure 3b and File S3) allowed us to assess how plastic each of them was for the different genetic stocks. Finally, the plot of how our measured individuals are distributed in the space defined by pairs of Dims revealed which variance components (G and/or T) are being separated along each Dim (supplementary File S3).

For both females and males, all eyespot traits seemed to contribute equivalently to defining Dim 1, which explained more than 70% of the variation in each respective dataset (70.8% for females and 77.7% for males; Figure 3a). Dim 1 was significantly affected by developmental temperature (df = 2, *p* < 2.2 × 10^−16^ for both females, F = 313.0, and males, F = 333.2), by genotype (df = 3, *p* < 2.2 × 10^−16^ for both females, F = 94.9, and males, F = 146.6), and by the interaction between these two factors (females: F = 19.4, df = 6, *p* < 2.2 × 10^−16^; males: F = 6.1, df = 6, *p* = 6.0 × 10^16^). WT was the least plastic genotype for Dim 1 (i.e., with smaller differences between temperature extremes, corresponding to a flatter reaction norm), and BE (eyespot size mutant) stood out once more for the intercept of Dim 1 reaction norm relative to all other lines (i.e., higher Dim 1 values relative to the other genotypes for both temperatures).

Dim 2 accounted for 13.4% of the variation in the female dataset and 7.4% of the variation in the male dataset, but it was similar for both datasets in that it largely contrasted black versus gold eyespot rings (loadings of opposite sign for the two colors) (Figure 3a). Two traits stood out in both datasets: the black area of eyespot e2 and the gold area of eyespot eP with loadings closer to zero, suggestive of little contribution to Dim 2. Dim 2 was significantly affected by genotype (df = 3, *p* < 2.2 × 10^−16^ for females, F = 258.7, and males, F = 140.5) and by genotype x temperature (females: F = 4.5, df = 6, *p* = 0.0002; males: F = 9.6, df = 6, *p* = 2.6 × 10^−9^) for both sexes. Differences between temperatures were marginally significant in the female dataset (F = 3.1, df = 2, *p* = 0.04) but not in the male dataset (F = 1.3, df = 2, *p* = 0.26). Dim 2 for Fr stood out from that of other genotypes, in both intercept and slope (Figure 3b).

Evaluating how the individuals we measured are distributed in the space defined by Dim 1 and Dim 2 (supplementary File S3), it becomes apparent that Dim 1, corresponding to the overall variation in eyespot size, mostly separated rearing temperatures, while Dim 2, contrasting gold and black rings, mostly separated genotypes. On the other hand, Dims 3 and 4, which together accounted for around 10% of the variation in the datasets and appeared to reflect contrasts between anterior versus posterior eyespots and fore- versus hindwing eyespots (Figure 3a), did not separate anything obvious in the datasets and had thermal reaction norms that were relatively flat and similar across genotypes (supplementary File S3).

### 3.3. Comparing Individuals Differing at Specific Pigmentation Loci

We wanted to test whether BE and Fr alleles, two alleles affecting different aspects of eyespot morphology (Figure 1 and Figure 2), had temperature-specific effects that resulted in differences in thermal reaction norms for eyespot color rings. We focused on the posterior eyespots on equivalent positions of the fore- and hindwings (eP and e5 in Figure 1); those are larger relative to the anterior eyespots (eA and e2) and include e5, which is the eyespot typically used in studies of wing pattern plasticity in *B. anynana*. We compared eyespots eP and e5 between siblings within each of the genetic stocks (Figure 4 and File S4). These sibling individuals differed in which allele they carried at the target pigmentation locus, but not in overall genetic background, allowing us to specifically test the effects of single allelic variants.

For both color rings in both eyespots and both sexes, there were significant GxT interactions (i.e., clear and significant differences in slope and shape of thermal reaction norms) between the four “genotypes” (Figure 4a; File S4). However, differences in plasticity between allelic variants were not seen in all cases. Rather, they depended on sex, eyespot, and/or color ring being analyzed. For the BE line, even though the reaction norms for siblings were clearly different in intercept, with both color rings being larger in BE relative to wild-type phenotype siblings, there were no significant GxT effects for any of the traits in males, nor for the golden ring of e5 in females. For the Fr line, reaction norms also differed in intercept (Fr relative to wild-type-looking siblings had smaller black rings and larger golden rings), while significant GxT effects were observed for eP (both color rings in males and black ring in females) but not for e5 (both color rings in both sexes). These results show that alleles with a large effect on pigmentation phenotypes can have temperature-specific effects and thus also contribute to variation in thermal plasticity in pigmentation. However, that was not observed for all traits analyzed. 

## 4. Discussion

We investigated thermal plasticity for the size of color rings of two eyespots on each of two wing surfaces in distinct *B. anynana* genetic lines differing in pigmentation phenotype (Figure 1). This allowed us to address two important properties of adaptive developmental plasticity, which can impact its evolution: (1) genetic variation for plasticity, corresponding to differences in reaction norms across genotypes, and (2) integration between plastic traits, corresponding to differences between reaction norms across individual eyespots or across their color rings. We know that plasticity evolves, and there are various examples in both natural and experimental populations [14,21,23,24,25], including for *Bicyclus* butterflies [22,26,45]. However, we still know surprisingly little about the genes that contribute to variation in plasticity and can fuel its evolution (discussed in [18]), as well as about the integration across plastic traits that might constrain their independent evolution (discussed in [49]).

By comparing thermal reaction norms between genetic lines, we found abundant examples of inter-genotype differences in plasticity. We documented significant GxT interactions, corresponding to inter-genotype differences in slope and/or curvature of thermal reaction norms, for the size of eyespot rings (Figure 2), as well as for the first principal component reflecting overall variation in eyespot size (Dim 1; Figure 3). There are reports of pervasive GxT interactions for various organisms and traits [57,58,59,60,61,62,63,64], but there are fewer examples in which the contribution of specific loci to variation in plasticity is tested. By focusing on plasticity for genetic lines featuring specific allelic variants known to affect pigmentation (Figure 1), we tested the hypothesis that genes contributing to pigmentation variation also contribute to variation in plasticity therein, which does not need to be the case (discussed in [18]). We found that individuals bearing mutant alleles of large effect on eyespot phenotype, namely BE and Fr, had reaction norms for eyespot ring size that could be significantly different from sibling individuals with wild-type alleles (Figure 4 and File S4). This means that the effects of these allelic variants on eyespot phenotype differ between temperatures or, conversely, that temperature effects on eyespot phenotype differ between genotypes. Such GxT interactions reflect differences in plasticity between allelic variants at the target loci and confirm the hypothesis that alleles affecting pigmentation can also affect thermal plasticity in pigmentation. This result, concerning mutant alleles of large effect on *B. anynana* eyespots (deliberately maintained in laboratory populations), contrasts with results from previous studies concerning alleles of more subtle phenotypic effect (segregating in large, outbred populations). In our study, we found that eyespot variants can differ in slope and curvature of thermal reaction norms. However, previous artificial selection experiments targeting quantitative variation, albeit able to alter eyespot size [38,39,40] and alter the intercept of reaction norms for eyespot size, were unable to alter the shape of those reaction norms [22,52]. This suggests that there was no segregating genetic variation for the slope or curvature of those thermal reaction norms. Low genetic variance for the shape of thermal reaction norms has been documented also in other systems [65], as has the apparent contrast between alleles of large and subtle effect. Studies in *Drosophila melanogaster* have shown that mutants of large phenotypic effect can affect thermal plasticity [58], including for body pigmentation [66,67,68,69]. In contrast, studies on quantitative variation revealed no overlap between QTLs contributing to intra-temperature variation and QTLs contributing to variation in thermal plasticity [17].

By analyzing thermal plasticity for the size of two color rings of distinct eyespots, we addressed the level of spatial compartmentalization, or of trait-specificity of plasticity and GxT effects. Specifically, we focused on two levels of the integration of plasticity: integration across pigmentation features (i.e., individual eyespots) and integration across components thereof (i.e., rings of color). *B. anynana* butterflies display serially repeated eyespots along the wing margins, each composed of white, black, and golden color rings produced in response to a concentration gradient of a signaling molecule diffusing from the presumptive eyespot center in developing pupal wings (cellular and genetic mechanisms reviewed in [31]). Eyespots tend to change in concert in relation to genetic (alleles of large and subtle effects [38,47,49,70]) and to environmental (notably, developmental temperature [30,43]) input. However, there is also some level of eyespot- and/or ring-specific responses, i.e., of spatial compartmentalization of the response to both types of input: (1) segregating genetic variation allowing independent evolutionary change in the overall size, but not the relative size of black and golden rings [71], of individual eyespots [39,40], and (2) differences between eyespots and between color rings [43] in response to external developmental temperature and/or to the internal ecdysone titers in pupae that mediate thermal plasticity in *B. anynana* [16,51,72]. This study addressed the interaction between genetic and environmental input by focusing on the effects of specific pigmentation genetic variants and developmental temperature. The results of the PCA summarizing axes of variation highlight patterns of integration between traits, a property which both reflects and potentially conditions their evolutionary history [49]. In particular, the second to fourth principal components, together accounting for over 15% of the variation in our datasets, contrasted individual eyespots and/or color rings (Figure 3a). Spatial compartmentalization of thermal plasticity in pigmentation has also been documented for other insects, including differences in reaction norms between melanization traits in *Pieris* butterflies [19], between abdominal segments in *D. melanogaster* [73], and between pigmentation components in *Drosophila* flies [74]. Moreover, previous studies have also found differences in QTLs responsible for plasticity in different body parts [17]. More generally, and even though thermal plasticity typically includes a series of traits changing in concert, often creating organism-wide phenotypes adjusted to conditions and collectively referred to as “plasticity syndromes” (see discussion in [50]), there are often trait-specific reaction norms. This is the case for the traits making up plasticity syndromes in *B. anynana* [72,75], for which reaction norms can evolve independently on each other [26]. Differences in reaction norms across plastic traits have also been documented for other species [76,77]. It is unclear how differences between traits that are part of the same plasticity syndrome come about. There might be differences in timing and/or level of sensitivity to environmental cues and/or to the hormones that convey information about those cues to developing tissues (shown also for *B. anynana* eyespots; e.g., [43,44]), as well as in downstream gene regulation (as shown for plasticity in *D. melanogaster* organ size; e.g., [78,79]). Further studies characterizing the molecular basis of plasticity, from whole-body hormonal signals to tissue-specific genetic mechanisms (e.g., [11,80]), as well as the link between the two (e.g., [9,81,82]), will be valuable in this respect.

Developmental plasticity is not only a mechanistically interesting and ecologically relevant property, but also one with tight connections to adaptive evolution (discussed in [18]). On the one hand, plasticity is heritable and can be adaptive, and, as such, it can and does evolve. On the other hand, plasticity has been argued to impact adaptive evolution, facilitating phenotypic and taxonomic diversification [12], and to affect population persistence and adaptation to environmental change, notably in insects [13]. In order to identify general principles about the evolution of plasticity, it is important to continue to add studies expanding on trait and taxonomic diversity. Crucially, it will be valuable to have studies investigating the combined effects of multiple environmental variables on multiple traits and in multiple genotypes, as this is the scenario that best represents natural populations and the challenges they face [13].

## Data Availability

Data supporting reported results can be found in supplementary File S1.

## Supplementary Materials

The following supporting information can be downloaded at: https://www.mdpi.com/article/10.3390/insects13111000/s1, File S1: Data on eyespot ring areas; File S2: Statistical analysis of T, G, and GxT effects on eyespot ring area; File S3: Extra analysis on principal components; File S4: Comparing color rings of eyespots eP and e5 between genotypes and allelic variants in males.
