# Supplementary material for: Developmental Plasticity in Butterfly Eyespot Mutants: Variation in Thermal Reaction Norms across Genotypes and Pigmentation Traits
Source: Insects. 2022 Oct 31;13(11):1000. doi: 10.3390/insects13111000 (PMC9699518; doi:10.3390/insects13111000)
Supplement: Supplementary file 1 [file insects-13-01000-s001.zip › File S2.pdf]

Table S2 - *Sample sizes.* Number of females (F) and males (M) measured for each of the target eyespots and wings from each genetic line (WT, BE, Fr, Choc) at each of the three temperatures (in the order: 19°C-23°C-27°C). For BE and Fr, the top row represents individuals of mutant phenotype (heterozygous at eyespot pigmentation locus) and the bottom row represents individuals of the wildtype phenotype (homozygous for the wildtype allele).

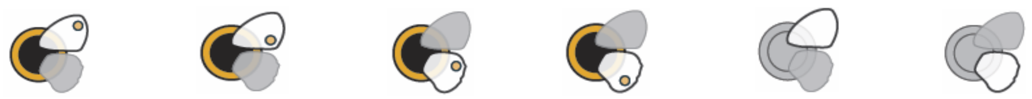

	**eA**	**eP**	**e2**	**e5**	**forewing**	**hindwing**
WT-F	30-30-30	30-30-30	27-30-30	27-30-30	32-30-31	29-30-30
WT-M	12-30-31	12-30-31	7-29-27	7-28-27	12-30-31	7-29-27
Choc-F	19-19-29	19-19-29	16-18-26	16-18-26	21-20-32	16-18-26
Choc-M	17-19-21	17-20-21	15-20-20	16-20-20	18-20-22	16-20-20
BE-F	9-7-22	10-7-22<br>5-5-7	8-6-19	9-6-19<br>4-5-5	10-7-22<br>5-5-7	9-6-19<br>4-5-5
BE-M	6-8-15	6-8-15<br>5-9-4	5-6-12	5-6-13<br>5-9-4	6-8-15<br>5-9-4	5-6-13<br>5-10-4
Fr-F	20-26-44	20-27-44<br>22-35-21	15-23-40	15-23-40<br>19-34-18	21-27-45<br>23-36-21	15-23-40<br>20-34-18
Fr-M	18-20-17	18-20-27<br>15-37-16	17-16-15	17-16-15<br>15-37-15	18-20-17<br>15-40-16	17-16-15<br>15-37-33

For each of the target eyespot rings (symbols), the tables display the results of the statistical tests for the effects of temperature (T), genotype (G), and GxT on **female eyespot ring area** using the corresponding wing area (forewing, fw, for eA and eP, and hindwing, hw, for e2 and e5) as covariate (model: ring_area ~ wing_area + Temperature * Genotype; top table), and pairwise comparisons (Tukey HSD where same letters in column "Groups" correspond to pairs of values not significantly different with alpha=0.01; bottom table).

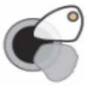
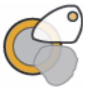
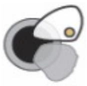
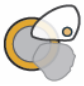

		**eA_black**			**eA_gold**			**eP_black**			**eP_gold**		
	Df	Dev	F	P	Dev	F	P	Dev	F	P	Dev	F	P
fw	1	0.31	8.37	0.004	0.02	0.95	0.32	0.02	3.69	0.05	0.07	10.99	0.001
G	3	2.72	24.33	5.58e-14	5.73	77.37	<2.2e-16	3.73	192.05	<2.2e-16	2.00	98.96	<2.2e-16
T	2	11.46	153.79	<2.2e-16	8.25	167.23	<2.2e-16	1.87	144.10	<2.2e-16	1.43	106.36	<2.2e-16
GxT	6	1.38	6.17	4.34e-06	0.83	5.64	1.53e-05	1.04	26.90	<2.2e-16	0.28	7.10	4.73e-07

**HSD**	Means	Groups	Means	Groups	Means	Groups	Means	Groups
WT_19	2.517	a	-0.124	d	0.798	d	5.822	ef
WT_23	2.492	a	0.130	bc	0.830	d	6.958	de
WT_27	2.424	b	0.183	b	0.843	cd	6.929	de
Choc_19	2.491	a	-0.323	e	0.701	e	4.937	f
Choc_23	2.468	ab	0.002	cd	0.835	d	7.249	cde
Choc_27	2.434	ab	0.042	bcd	0.811	d	7.903	cd
Fr_19	2.458	ab	-0.047	d	0.410	f	6.481	def
Fr_23	2.460	ab	0.197	b	0.684	e	8.748	cd
Fr_27	2.419	b	0.484	a	0.806	d	10.910	b
BE_19	2.483	ab	-0.124	de	0.954	bc	9.254	bc
BE_23	2.513	a	0.102	bcd	1.008	ab	9.014	bcd
BE_27	2.445	ab	0.386	a	1.105	a	12.720	a

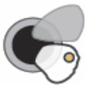
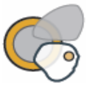
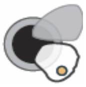
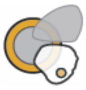

		**e2_black**			**e2_gold**			**e5_black**			**e5_gold**		
	Df	Dev	F	P	Dev	F	P	Dev	F	P	Dev	F	P
hw	1	0.42	8.36	0.004	0.36	29.72	1.21e-07	0.06	4.21	0.04	0.62	46.97	5.74e-11
G	3	5.18	33.80	<2.2e-16	4.28	117.81	<2.2e-16	2.13	48.43	<2.2e-16	3.96	99.37	<2.2e-16
T	2	19.22	188.20	<2.2e-16	5.30	218.76	<2.2e-16	6.81	232.25	<2.2e-16	6.13	230.59	<2.2e-16
GxT	6	2.30	7.53	1.99e-07	0.95	13.19	6.14e-13	0.67	7.63	1.56e-07	0.50	6.37	2.95e-06

**HSD**	Means	Groups	Means	Groups	Means	Groups	Means	Groups
WT_19	-0.769	d	-0.445	e	0.214	c	0.068	e
WT_23	-0.420	abc	-0.267	c	0.406	b	0.243	cd
WT_27	-0.317	ab	-0.263	c	0.478	b	0.335	bc
Choc_19	-1.259	e	-0.656	f	0.001	d	-0.110	f
Choc_23	-0.724	cd	-0.402	de	0.308	bc	0.174	de
Choc_27	-0.528	bc	-0.286	cd	0.379	b	0.289	bcd
Fr_19	-1.248	e	-0.485	e	-0.172	e	0.103	e
Fr_23	-0.375	ab	-0.135	bc	0.242	c	0.414	b
Fr_27	-0.218	a	0.063	a	0.407	b	0.594	a
BE_19	-0.881	de	-0.459	e	0.195	c	0.048	ef
BE_23	-0.376	abc	-0.128	bc	0.494	ab	0.392	bc
BE_27	-0.274	ab	-0.008	ab	0.658	a	0.591	a

For each of the target eyespot rings (symbols), the tables display the results of the statistical tests for the effects of temperature (T), genotype (G), and GxT on **male eyespot ring area** using the corresponding wing area (forewing, fw, for eA and eP, and hindwing, hw, for e2 and e5) as covariate (model: ring_area ~ wing_area + Temperature * Genotype; top table), and pairwise comparisons (Tukey HSD where same letters in column "Groups" correspond to pairs of values not significantly different with alpha=0.01; bottom table).

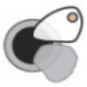
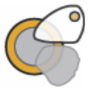
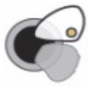
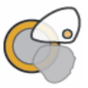

		**eA_black**			**eA_gold**			**eP_black**			**eP_gold**		
	Df	Dev	F	P	Dev	F	P	Dev	F	P	Dev	F	P
fw	1	0.01	0.20	0.65	0.01	0.04	0.83	43.37	47.16	7.89e-11	0.10	8.84	0.003
G	3	24.54	89.39	<2.2e-16	2.59	38.30	<2e-16	690.30	250.20	<2.2e-16	1.57	42.42	<2.2e-16
T	2	25.79	140.93	<2.2e-16	6.47	143.44	<2e-16	287.62	156.37	<2.2e-16	1.57	42.42	<2.2e-16
GxT	6	1.68	3.07	0.006	0.35	2.59	0.01	17.22	3.12	0.006	0.08	1.08	0.37

**HSD**	Means	Groups	Means	Groups	Means	Groups	Means	Groups
WT_19	0.207	g	2.566	f	0.355	e	2.566	e
WT_23	0.697	ef	3.720	cd	0.594	d	3.720	de
WT_27	1.133	bc	4.107	b	0.657	cd	4.107	de
Choc_19	0.240	g	2.484	ef	0.389	e	2.484	e
Choc_23	0.758	def	4.093	bcd	0.646	cd	4.093	de
Choc_27	1.054	bcd	4.467	bc	0.718	c	4.467	cd
Fr_19	0.190	g	2.949	de	0.083	f	2.949	e
Fr_23	0.419	fg	3.792	bcd	0.340	e	3.792	de
Fr_27	0.890	cde	5.508	a	0.618	cd	5.508	bc
BE_19	0.665	efg	4.250	bcd	0.770	bc	4.250	cde
BE_23	1.426	b	6.468	ab	0.948	ab	6.468	ab
BE_27	2.052	a	7.911	a	0.979	a	7.911	a

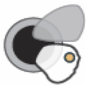
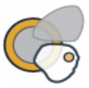
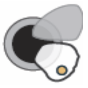
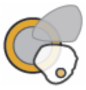

		**e2_black**			**e2_gold**			**e5_black**			**e5_gold**		
	Df	Dev	F	P	Dev	F	P	Dev	F	P	Dev	F	P
hw	1	0.17	6.38	0.01	0.03	2.41	0.12	0.01	0.25	0.61	0.05	6.23	0.01
G	3	4.66	57.16	<2e-16	1.83	42.49	<2e-16	3.62	85.83	<2.2e-16	1.78	68.51	<2e-16
T	2	7.01	128.97	<2e-16	3.78	131.92	<2e-16	6.13	218.05	<2.2e-16	3.24	186.45	<2e-16
GxT	6	0.42	2.62	0.01	0.24	2.86	0.01	0.28	3.32	0.003	0.10	1.96	0.07

**HSD**	Means	Groups	Means	Groups	Means	Groups	Means	Groups
WT_19	0.069	e	-0.236	de	0.214	e	-0.194	g
WT_23	0.312	d	-0.284	bc	0.406	d	0.099	ef
WT_27	0.530	b	0.530	d	0.478	bc	0.198	cd
Choc_19	0.075	e	-0.663	e	0.001	e	-0.134	g
Choc_23	0.314	d	-0.280	bc	0.308	cd	0.140	def
Choc_27	0.519	bc	-0.245	b	0.379	bc	0.188	cde
Fr_19	0.101	de	0.101	cd	-0.213	e	0.041	f
Fr_23	0.318	cd	0.318	b	0.140	d	0.265	bc
Fr_27	0.637	b	-0.026	a	0.273	cd	0.359	ab
BE_19	0.300	de	-0.308	bcd	0.315	bcd	0.160	cdef
BE_23	0.668	b	-0.095	ab	0.553	ab	0.341	abc
BE_27	1.112	a	0.075	a	0.590	a	0.476	a

For each of the target eyespot rings (symbols), the tables display the results of the statistical tests for the effects of temperature (T), genotype (G), and GxT on **wing area** (model: wing_area ~ Temperature * Genotype; top table), and pairwise comparisons (Tukey HSD where same letters in column "Groups" correspond to pairs of values not significantly different with alpha=0.01; bottom table). Analyses were run separately for females **(a)** and males **(b)**.

a) females

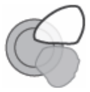

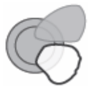

		forewing			hindwing		
	Df	Dev	F	P	Dev	F	P
G	3	11695	3.83	0.01	34993	8.92	1.23e-05
T	2	33935	16.68	1.40e-07	70406	26.93	2.62e-11
GxT	6	17024	2.79	0.01	8162	1.04	0.39

HSD	forewing		hindwing	
	Means	Groups	Means	Groups
WT_19	322.5	ab	384.8	a
WT_23	317.6	ab	368.1	a
WT_27	314.7	ab	356.3	abc
Choc_19	338.8	a	365.9	ab
Choc_23	341.6	a	351.8	abc
Choc_27	296.6	b	315.2	c
Fr_19	348.4	a	364.7	ab
Fr_23	336.7	a	336.7	ab
Fr_27	323.3	ab	323.3	bc
BE_19	351.5	a	351.5	a
BE_23	326	ab	326	abc
BE_27	321	ab	321	bc

b) males

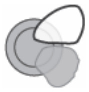

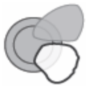

		forewing			hindwing		
	Df	Dev	F	P	Dev	F	P
G	3	8026	3.32	0.02	0.01	1.76	0.15
T	2	35218	21.89	2.4e-09	0.13	25.71	1.52e-10
GxT	6	3798	0.78	0.58	0.01	1.18	0.31

HSD	forewing		hindwing	
	Means	Groups	Means	Groups
WT_19	290	ab	2.517	a
WT_23	297	a	2.492	a
WT_27	262.4	b	2.424	b
Choc_19	291.9	ab	2.491	a
Choc_23	283.7	ab	2.468	ab
Choc_27	261.8	b	2.434	ab
Fr_19	256.2	ab	2.458	ab
Fr_23	275.3	ab	2.460	ab
Fr_27	256.2	b	2.419	b
BE_19	289.5	ab	2.483	ab
BE_23	309.9	a	2.513	a
BE_27	279.8	ab	2.445	ab
