# Supplementary material for: Developmental Plasticity in Butterfly Eyespot Mutants: Variation in Thermal Reaction Norms across Genotypes and Pigmentation Traits
Source: Insects. 2022 Oct 31;13(11):1000. doi: 10.3390/insects13111000 (PMC9699518; doi:10.3390/insects13111000)
Supplement: Supplementary file 1 [file insects-13-01000-s001.zip › File S3.pdf]

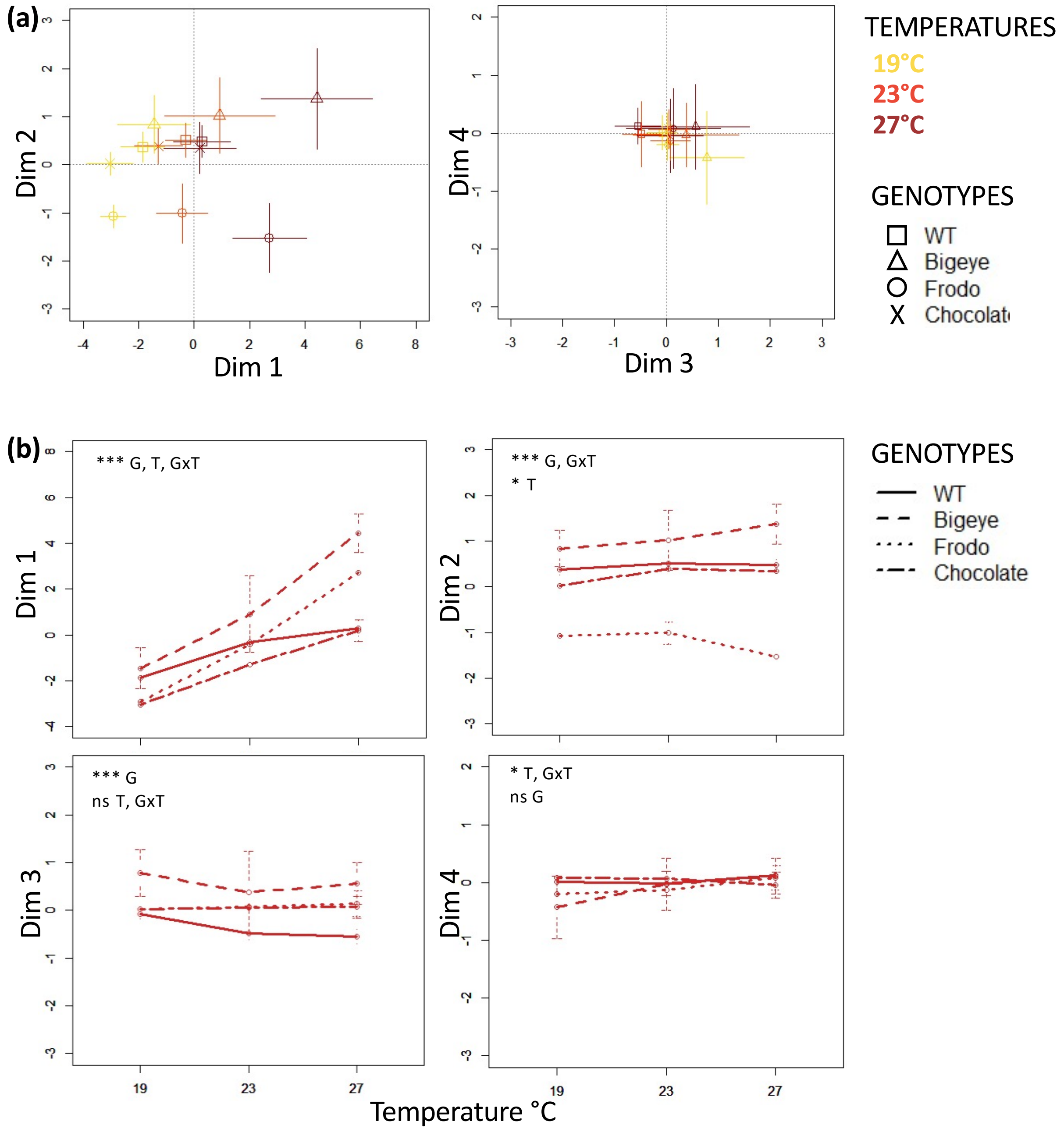
(a)
Dim 1
Dim 2
Dim 3
Dim 4
TEMPERATURES
19°C
23°C
27°C
GENOTYPES
WT
Bigeye
Frodo
Chocolate
(b)
*** G, T, GxT
*** G, GxT
* T
*** G
ns T, GxT
* T, GxT
ns G
GENOTYPES
WT
Bigeye
Frodo
Chocolate
Temperature °C

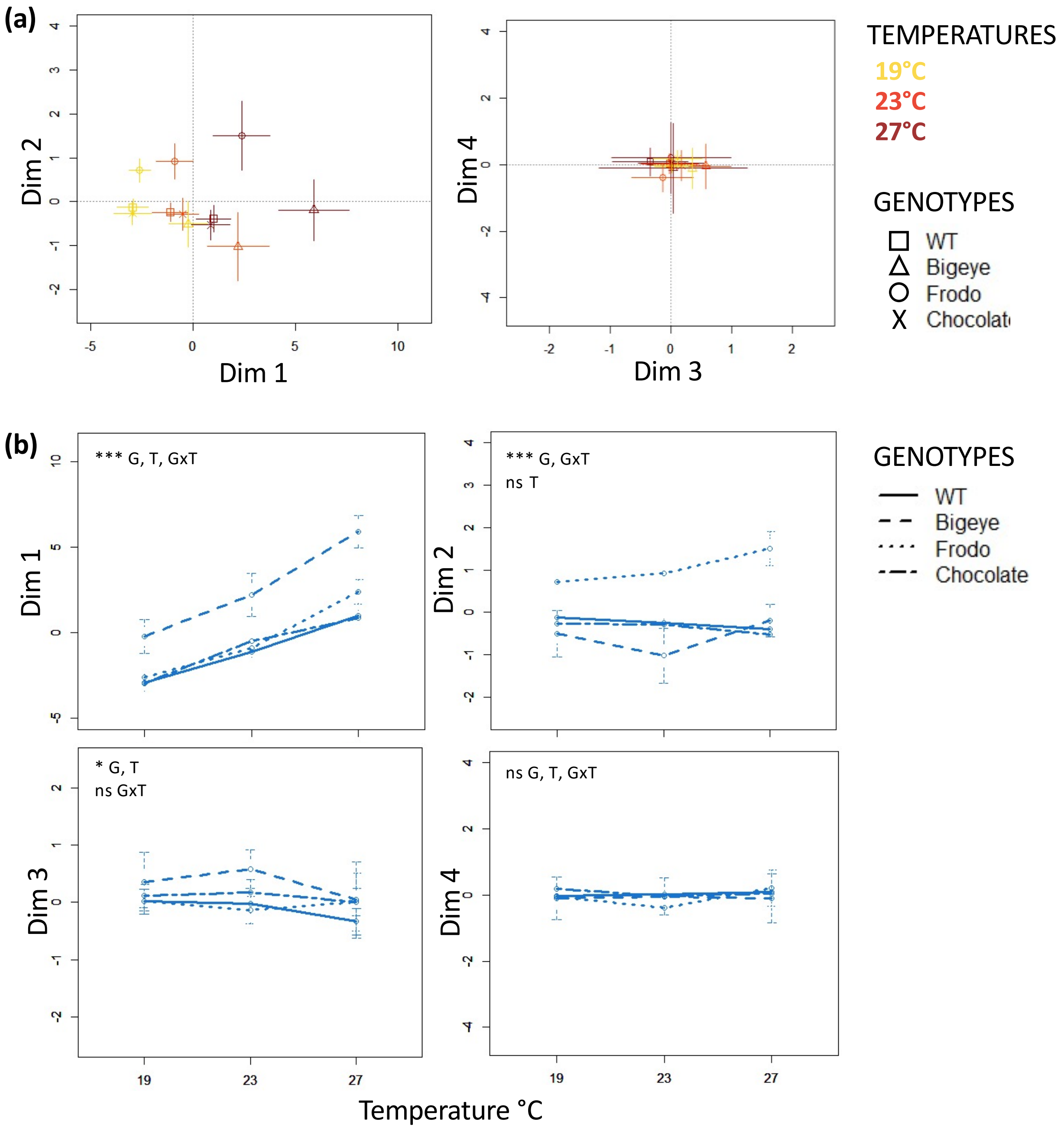
(a)
Dim 2
Dim 1
Dim 4
Dim 3
TEMPERATURES
19°C
23°C
27°C
GENOTYPES
WT
Bigeye
Frodo
(b)
*** G, T, GxT
Dim 1
*** G, GxT
ns T
Dim 2
* G, T
ns GxT
Dim 3
ns G, T, GxT
Dim 4
GENOTYPES
WT
Bigeye
Frodo
Chocolate
Temperature °C

Figure S3 - Principal component analysis (PCA) of eyespot ring area for different temperatures and genotypes. **(a)** Distribution of mean values per temperature and genotype (legends to the right) for the four main principal components, Dims 1-4, showing what aspects of variation each Dim separates. **(b)** Mean values along Dims 1-4 (with bars representing standard deviation) as a function of developmental temperature (19, 23, or 27°C) for each of the four genotypes (WT, Fr, BE and Choc in different line styles; legend on bottom right corner) in females (S3i) and males (S3ii). We tested for the effect of temperature (T) and genotype (G) on each Dim (see Material and Methods). Statistical significance of effects of G, T, and GxT is represented with ns for non-significant ($P>0.05$), * for $P<0.05$, ** for $P<0.01$, *** $P<0.001$.

Results of the statistical tests for the effects of temperature (T), genotype (G), and GxT on each of the first four **Principal Components**, Dims 1-4, (model: Dim~Genotype*Temperature; top table), and pairwise comparisons (Tukey HSD where same letters in column "Groups" correspond to pairs of values not significantly different with alpha=0.01; bottom table). Analyses were run separately for females **(a)** and males **(b)**.

a) females

		Dim 1			Dim 2			Dim 3			Dim 4		
	Df	Dev	F	P	Dev	F	P	Dev	F	P	Dev	F	P
G	3	377.17	94.91	<2.2e-16	233.26	258.73	<2.2e-16	29.94	27.66	8.19e-16	0.53	0.69	0.55
T	2	829.11	312.96	<2.2e-16	1.88	3.13	0.04	1.58	2.19	0.11	1.41	5.51	0.01
GxT	6	154.04	19.38	<2.2e-16	8.08	4.48	0.0002	3.78	1.74	0.10	2.48	3.21	0.02

HSD	Dim 1		Dim 2		Dim 3		Dim 4	
	Means	Groups	Means	Groups	Means	Groups	Means	Groups
WT_19	-1.852	ef	0.370	bc	-0.082	bc	0.008	a
WT_23	-0.302	cd	0.517	bc	-0.476	bc	-0.013	a
WT_27	0.277	c	0.483	bc	-0.549	c	0.124	a
Choc_19	-3.036	f	0.024	c	0.022	abc	0.088	a
Choc_23	-1.290	de	0.388	bc	0.054	abc	0.069	a
Choc_27	0.204	c	0.350	bc	0.071	ab	-0.049	a
Fr_19	-2.917	f	-1.068	de	0.023	abc	-0.198	a
Fr_23	-0.431	cd	-1.008	d	0.082	ab	-0.129	a
Fr_27	2.722	b	-1.520	e	0.141	ab	0.081	a
BE_19	-1.447	def	0.841	ab	0.779	a	-0.425	a
BE_23	0.914	c	1.025	ab	0.379	ab	-0.029	a
BE_27	4.427	a	1.377	a	0.565	a	0.112	a

b) males

		Dim 1			Dim 2			Dim 3			Dim 4		
	Df	Dev	F	P	Dev[a]	F	P	Dev[a]	F	P	Dev	F	P
G	3	440.34	146.62	<2.2e-16	-	140.50	<2.2e-16	-	3.11	0.02	1.10	1.06	0.36
T	2	667.03	333.16	<2.2e-16	-	1.32	0.26	-	3.43	0.03	1.33	1.93	0.14
GxT	6	36.93	6.14	6.02e-06	-	9.60	2.627e-09	-	0.96	0.45	2.59	1.24	0.28

[a]: For Dims 2 and 3 we used the model: ((Dim+3) ^lambda - 1)/lambda ~Genotype*Temperature. With this model we have no Deviance.

HSD	Dim 1		Dim 2		Dim 3	
	Means	Groups	Means	Groups	Means	Groups
WT_19	-2.929	f	1.522	c	1.618	a
WT_23	-1.103	e	1.441	c	1.592	a
WT_27	1.015	cd	1.336	c	1.358	a
Choc_19	-2.943	f	1.424	c	1.682	a
Choc_23	-0.501	e	1.407	c	1.725	a
Choc_27	0.867	cd	1.231	cd	1.606	a
Fr_19	-2.596	f	2.082	b	1.619	a
Fr_23	-0.892	e	2.210	ab	1.507	a
Fr_27	2.378	b	2.565	cd	1.582	a
BE_19	-0.230	de	1.241	cd	1.846	a
BE_23	2.210	bc	0.825	d	1.995	a
BE_27	5.903	a	1.456	c	1.572	a
