# Supplementary material for: Developmental Plasticity in Butterfly Eyespot Mutants: Variation in Thermal Reaction Norms across Genotypes and Pigmentation Traits
Source: Insects. 2022 Oct 31;13(11):1000. doi: 10.3390/insects13111000 (PMC9699518; doi:10.3390/insects13111000)
Supplement: Supplementary file 1 [file insects-13-01000-s001.zip › File S4.pdf]

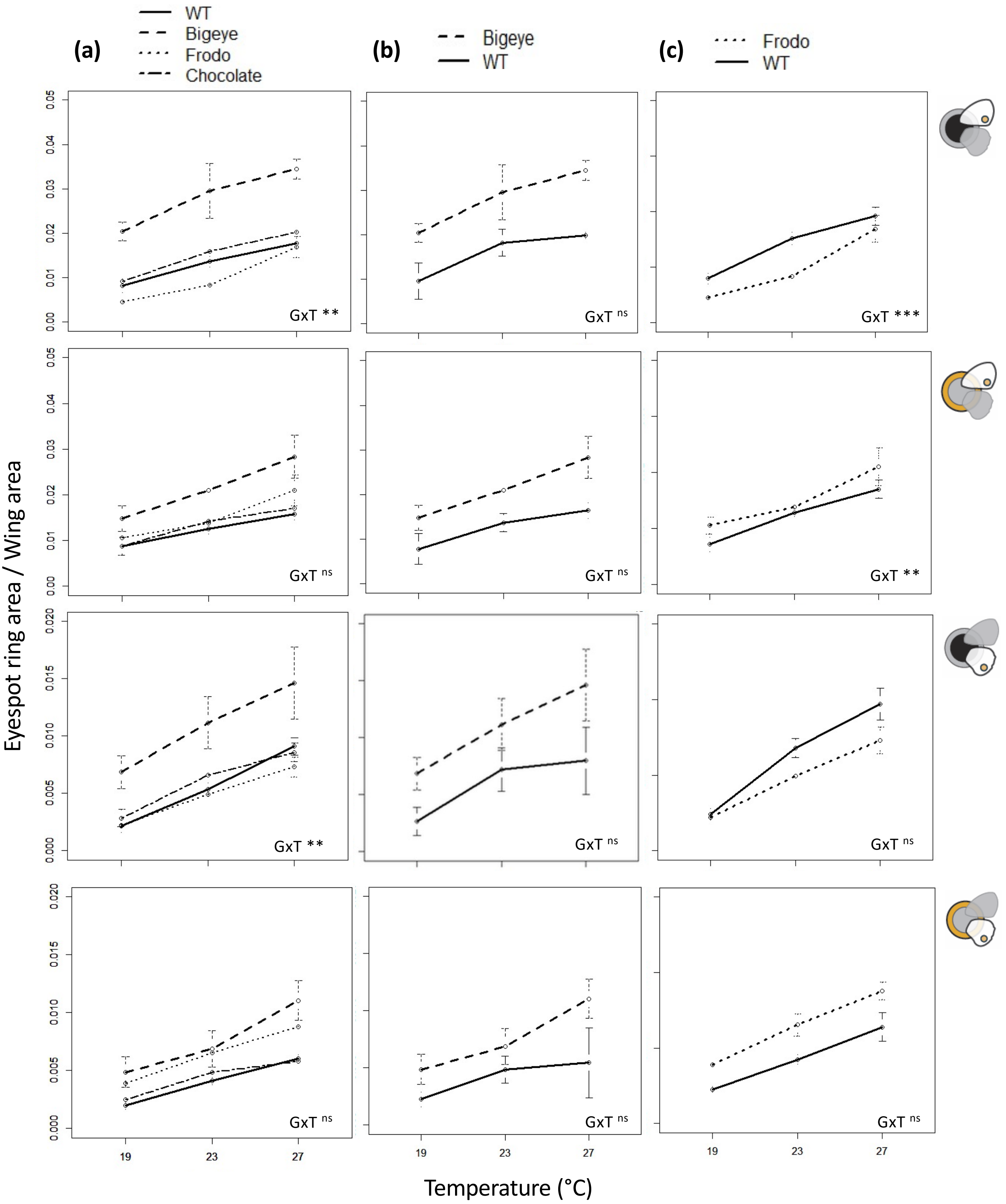

Figure S4 - *Thermal reaction norms for eyespot ring area for butterflies differing at specific pigmentation loci.* Means and standard deviations for eyespot ring areas (symbols to the right) relative to corresponding wing areas, across developmental temperatures in males from different genetic lines **(a)**, and allelic variants for the BE **(b)** and Fr **(c)**, which compare "mutant" (heterozygous at respective locus) and "wildtype" (homozygous for wildtype allele) siblings. We tested for the effect of temperature (T) and genotype (G) on eyespot ring area using wing area as covariate (see Material and Methods). Statistical significance for GxT interactions displayed as: ns (non-significant) P>0.05, *P<0.05, ** P<0.01, *** P<0.001.
